# Spark Plasma Sintering of Pure Titanium: Microstructure and Mechanical Characteristics

**DOI:** 10.3390/ma17143469

**Published:** 2024-07-13

**Authors:** Satyavan Digole, Sanoj Karki, Manoj Mugale, Amit Choudhari, Rajeev Kumar Gupta, Tushar Borkar

**Affiliations:** 1 Department of Mechanical Engineering, Cleveland State University, Cleveland, OH 44115, USA; 2 Department of Materials Science and Engineering, North Carolina State University, Raleigh, NC 27695, USA

**Keywords:** spark plasma sintering, titanium, grain size, relative density, mechanical property

## Abstract

The versatility of titanium (Ti) allows it to be employed in various industries, from aerospace engineering to medical technology, highlighting its significance in modern manufacturing and engineering processes. Spark plasma sintering (SPS) is currently being explored to enhance its properties further and broaden its application range. The current study focuses on exploring and optimizing the effect of SPS temperature (800, 900, 1000, 1100, 1200, and 1400 °C) on pure Ti sintered at 60 MPa in a controlled argon environment with a dwell time of 5 min. All the prepared samples were highly dense with a relative density above 99%, but exhibited significant variations in grain size (10 to 57 µm), tensile yield strength (488 to 700 MPa), ultimate tensile strength (597 to 792 MPa), and ductility (4 to 7%). A microstructural investigation was performed using XRD, SEM, and EDS to predict the influence of sintering temperature on the formation of different phases. The XRD patterns of all sintered samples showed the presence of single-phase α-Ti with hexagonally close-packed Ti. This work is a step forward in optimizing SPS-processed Ti’s physical and mechanical properties for enhanced structural and biomedical applications.

## 1. Introduction

Lightweight materials are crucial for aerospace applications due to their ability to enhance system energy efficiency [1]. Small weight reductions in the space and aerospace industry result in higher cost savings than in the automotive industry, highlighting the demand for lighter metals [2]. Weight reduction can be achieved by density reduction, assuming that the geometrical dimensions of the component remain unchanged, which underscores the significance of lighter materials. In comparison with steel, nickel, and aluminum alloys, titanium (Ti) alloys are widely used in the aerospace industry due to their excellent corrosion resistance, high fatigue resistance, high melting point, stable properties at high temperatures, and high strength-to-weight ratio [3]. The aviation industry significantly can reduce the thrust-to-weight ratio of aviation engines by replacing nickel-based superalloys with Ti alloys [4]. Different composites, metals, and polymers are used as biometals, from which Ti and its alloys are commonly used in orthopedic and dental implants due to their effectiveness [5,6,7,8]. Its biocompatibility, osseointegration, low density, mechanical properties, and corrosion resistance are the properties that make Ti and its alloys the first choice for biometals for orthopedic applications [9,10,11,12,13].

A critical barrier in using Ti components is the cost that comes from raw materials and processes [3]. There are different routes for processing titanium, including powder metallurgy (PM), casting, and machining [14]. However, Ti’s high reactivity and low thermal conductivity make it difficult to cast and machine; therefore; PM is the preferred route for processing Ti components [15,16,17]. Hot pressing (HP) is the PM technique in which temperature and uniaxial pressure are raised at the same time to obtain non-porous components. Punches and dies are used in HP to compact the free powder, whereas heating is applied through high-temperature resistance heating. Conventional sintering limits the heating–cooling rates, current supply, and mechanical pressure that can be overcome in field-assisted sintering, also called the spark plasma sintering technique [18]. Spark plasma sintering (SPS) is the most effective technique for overcoming the above-mentioned issues. A high-energy pulsed current during SPS results in the pressurized impact of plasma, causing the localized melting and neck formation of powder particles, which leads to the breakage of oxide layers within the contacted particle and increases mass transport. The controllable pressure, temperature, and short processing time during SPS limit unwanted grain growth, which improves mechanical properties [19,20,21,22,23,24].

The SPS process parameters can affect the characteristics of fabricated pure Ti. Asl et al. [25] used hydride–dehydride pure Ti powder (<75 μm) for SPS processing at five different temperatures, ranging from 750 to 1350 °C, under 50 MPa pressure in a vacuum for 5 min dwell time. The influence of densification, α and β phase formation, and grain size on the mechanical characteristics for different processing temperatures were analyzed, and optimum properties (hardness: 391 HV, bending strength > 2000 MPa, and tensile strength: 500 MPa) were reported at 1200 °C sintering temperature. Also, the nanoindentation technique was used to calculate the correlation among the formed phase’s elastic modulus, hardness, and contact stiffness. Miklaszewski et al. [26] analyzed the effect of the SPS sintering temperature, ranging from 800 to 1500 °C, on the microstructure and mechanical properties of sintered pure Ti powder (325 mesh). Equiaxed polyhedral Ti (α) grains were observed below the β-transus temperature, whereas Ti (α) laths were observed above the β-transus temperature. Alterations in the microstructure and grain coarsening at higher sintering temperatures resulted in the degradation of mechanical properties. Maximum tensile and compressive strengths were reported at a sintering temperature of 900 °C. Chaudhari et al. [27] performed one-stage and three-stage SPS consolidation for pure Ti powder (45 μm). A maximum SPS temperature and pressure of 1300 °C and 50 MPa in a 6 Pa vacuum for a dwell time of 10 min were considered for one-stage sintering. Three different sintering temperatures (600, 1100, and 1300 °C) with different heating rates (100 °C/minutes and 25 °C/minute) and holding times (5 min and 10 min) were considered for three-stage SPS processing. The mechanical properties and densification observed at three-stage sintering were effective because of the lower percentage of β phase formation than in one-stage sintering. Also, different phase morphologies were observed at different locations, predominantly affecting the properties of the consolidated samples. Along with the SPS parameters, the presence of impurities in pure Ti powder had an influence on the microstructure and mechanical properties of the processed sample. Zadra et al. [28] processed two different grades of pure Ti powder (grade 1 and grade 3) through SPS at a temperature range of 700–1050 °C, pressure of 60 MPa, and dwell time of 5 min in a controlled vacuum. Density, microstructure, hardness, tensile testing, and fractography analyses were performed to investigate the effect of the SPS process on sample characteristics. Grade 3 titanium exhibited better properties than grade 1 titanium when sintered both below and above the hcp (α) to bcc (β) transition temperature due to its higher oxygen content. While the grade 3 sample exhibited a significant reduction in ductility and a distinct plate-like fracture pattern when sintered over the α to β transition temperature, grade 1 only exhibited slightly altered mechanical properties (a slight reduction in strength). Superior mechanical properties were observed when Ti powders of different grades were sintered in an α-phase field. Motsi et al. [29] sintered two different pure Ti powders: 1. Ti(S): Higher amounts of oxygen with an average particle size of 25.9 μm. 2. Ti(L): Higher amounts of hydrogen with an average particle size of 35.9 μm. SPS was performed on both powders with a temperature range of 550–900 °C and a pressure range of 25–75 MPa. Different percentages of impurities led to the formation of different microstructures in sintered pellets. The presence of oxygen in Ti(S) and hydrogen in Ti(L) led to the formation of α needle and α lamellar microstructures, respectively. A higher hardness of 340 HV was reported at 800 °C under 25 MPa pressure for Ti(S) due to the needle-like microstructure and smaller particle size than that of Ti(L).

Although some research has focused on process optimization to improve the characteristics of pure titanium, less investigation into the effect of SPS temperature on microstructural changes and tensile characteristics has been found. Furthermore, to achieve the desired characteristics of any Ti matrix-based composite process through SPS, it is crucial to fix the appropriate SPS temperature to achieve better matrix properties. However, it is important to investigate and understand the effects of SPS processing parameters on the relative density, grain size, hardness, phase formation, and mechanical properties of pure Ti. This work primarily investigates the influence of SPS temperature on the microstructure and mechanical properties of pure titanium. The aim is to achieve a better intellectual understanding of the role of SPS temperature in enhancing the overall performance of titanium and potentially expanding its application areas.

## 2. Materials and Methods

**Figure 1:**
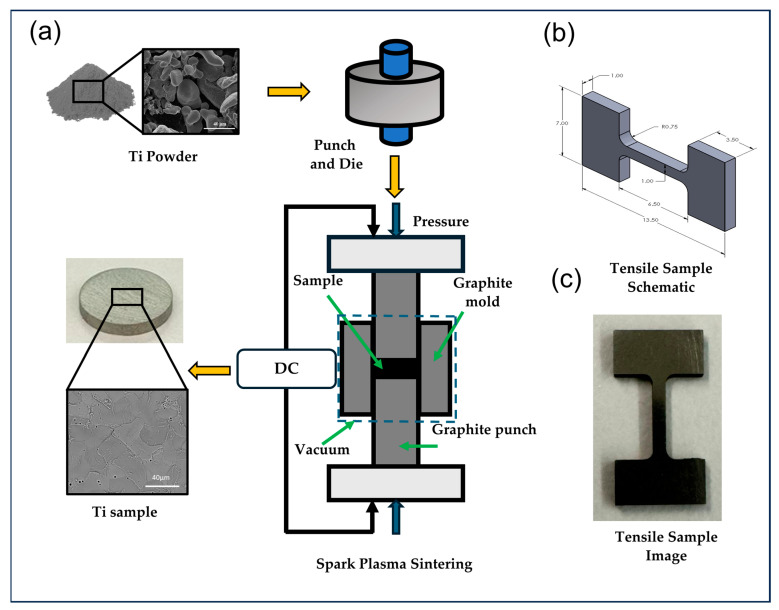
(**a**) SPS Sample preparation process layout, (**b**) tensile sample schematic, and (**c**) tensile sample image.

Pure Ti powder manufactured by the hydride–dehydride process with a purity greater than 99.7% and particle size less than 20 µm was purchased from Atlantic Equipment Engineers (Upper Saddle River, NJ, USA). An amount of 5 g of loose Ti powder was initially compacted using a graphite die and punch under 5 MPa pressure. The powders were shaped into a bulk form using SPS 10–3 spark plasma sintering (SPS) by Thermal Technologies LLC (Santa Rosa, CA, USA). SPS of pure Ti powders was performed at different temperatures (800, 900, 1000, 1100, 1200, and 1400 °C) under 60 MPa pressure under a controlled argon atmosphere for 5 min of dwell/holding time. The heating rate of 100 °C/min was maintained. The sintered samples were in the form of a thin disc with a 20 mm diameter and 2.5 mm thickness. The sample preparation process is shown in Figure 1a. The processed samples were then polished on a BUEHLER AutoMet^TM^ 250 Grinder–Polisher (Lake Bluff, IL, USA) using silicon carbide papers (120, 240, 400, 600, 800, and 1200 grit size). The final polishing was performed using a micro cloth with a mixture of colloidal silica and attack polishing agent (50 mL colloidal silica, 10 mL H_2_O_2_ (30%), and 5 mL Kroll’s reagent) [30]. The samples were then cleaned with ethanol in an ultrasonic bath to eliminate any additional small particles/debris and achieve the desired surface finish.

Microstructure characterization and phase morphology investigation were performed by scanning electron microscopy (SEM) using Inspect F50 (FEI, now Thermo Fisher Scientific, Hillsboro, OR, USA). Grain size analysis was performed using ImageJ software version 1.54, and the average of 100 grain size measurements was reported. The actual bulk density of the sample was estimated from the Archimedes principle with deionized (DI) water as a working medium. The relative density is the ratio of the measured bulk density to theoretical density (4.506 gm/cm^3^) and was calculated for all the samples accordingly. X-ray diffraction (XRD) analysis and energy dispersive X-ray spectroscopy (EDS) were performed to identify different phases present in the specimen. A microhardness Tester (Wilson VH1202 BUEHLER Lake Bluff, IL, USA) with a Vickers indenter was used to measure the hardness of all sintered samples. An average of ten hardness readings were taken at different locations on the sintered samples. Tensile testing was performed using a Kammrath & Weiss mini tensile machine (An der Silberkuhle 1, Schwerte, Germany) with a dog-bone-shaped sample of 5 mm gauge length, 1 mm width, and 1 mm thickness cut using a Sodick VN400Q wire electrical discharge machine (EDM) (Schaumburg, IL, USA). A schematic and an actual image of the tensile sample are shown in Figure 1b,c. Two samples were considered for tensile testing to confirm the consistency of the result. An engineering stress–strain curve was created using load extension data from tensile testing by computing the yield strength (YS), ultimate tensile strength (UTS), and elongation up to failure.

All the experiments were performed randomly considering a sample size equal to two. Statistical analysis was conducted using the F-test in Minitab 21 software (one-way ANOVA), with a *p*-value less than 0.05 considered statistically significant. Further Tukey tests were performed to identify which treatment level mean was significantly different from the other level means. The data from multiple independent experiments are displayed as the mean ± standard error.

## 3. Results and Discussion

### 3.1. Structural Analysis

**Figure 2:**
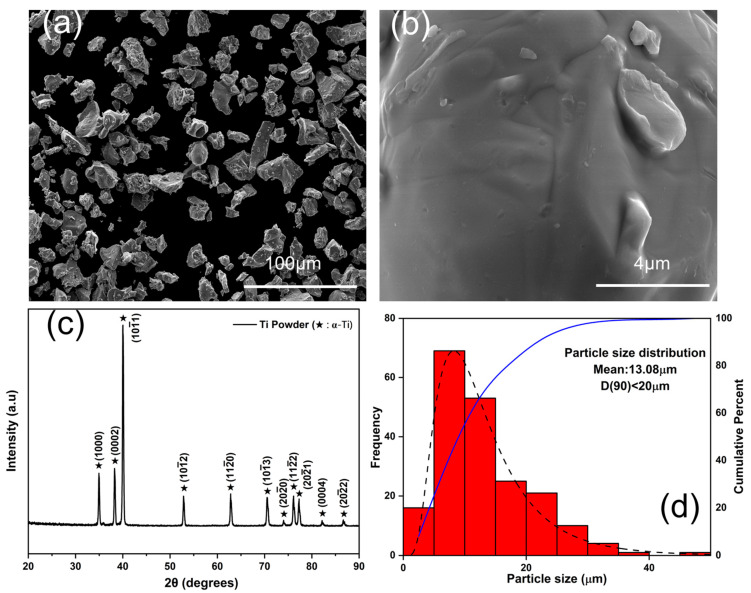
SEM images of Ti powder particles at (**a**) lower magnification, (**b**) higher magnification, (**c**) XRD plot for pure Ti powder, and (**d**) Ti particle size distribution.

Scanning electron microscopy (SEM) images of the Ti powder are shown in Figure 2a, which display coarse and fine irregularly shaped morphology powder particles. The clean surface of the particle in Figure 2b confirms the absence of any impurity within the powder. The XRD pattern for Ti powder in Figure 2c shows only α-Ti peaks, which further confirm its purity. Figure 2d presents the particle size distribution for Ti powder, with more than 90% of particles having a size of <20 µm, which is in good agreement with the standard particle size specifications provided by the supplier.

**Figure 3:**
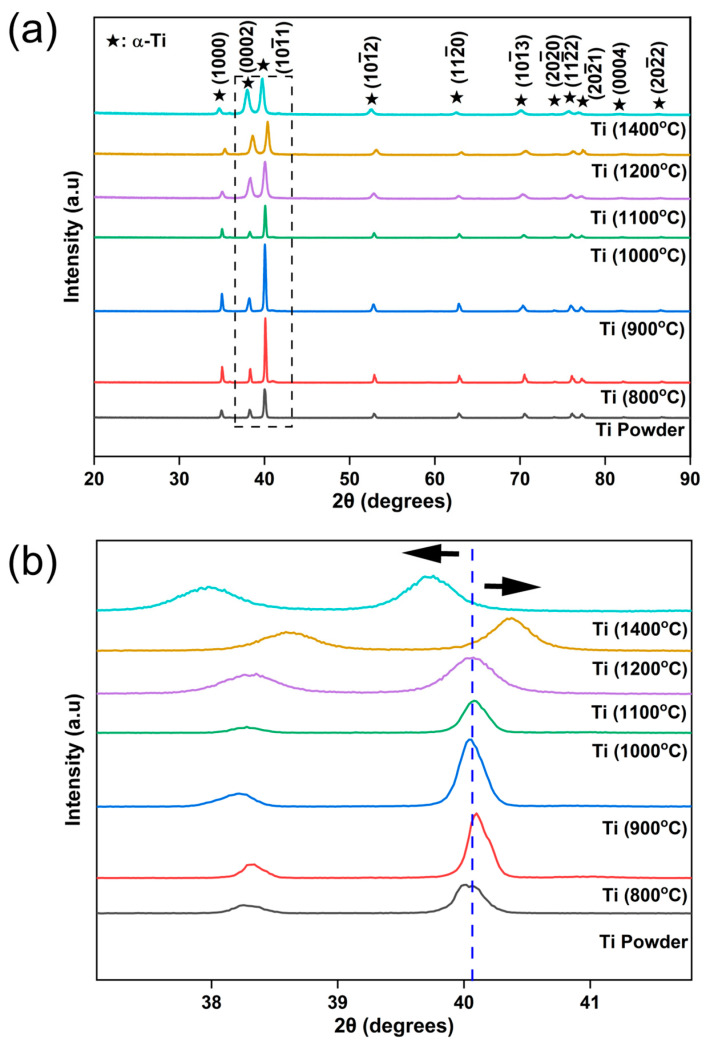
XRD plot of (**a**) SPS sintered sample and (**b**) enlarged view with arrow showing peak shift towards higher and lower 2θ as a function of SPS temperature.

XRD patterns of the pure Ti powder and sintered pure Ti at 800, 900, 1000, 1100, 1200, and 1400 °C at a constant sintering pressure of 60 MPa are shown in Figure 3a. The XRD patterns of all samples showed strong peaks related to α-Ti associated with (1000), (0002), (10$\bar{1}$1), (10$\bar{1}$2), (11$\bar{2}$0), (10$\bar{1}$3), (20$\bar{2}$0), (11$\bar{2}$2), (20$\bar{2}$1), (0004) and (20$\bar{2}$2) planes, similar to that of pure Ti powder, suggesting that there was no specific effect of temperature on phase transformation. An enlarged image of the peak corresponding to the (10$\bar{1}$1) plane of the Ti powder and Ti samples sintered at different temperatures is shown in Figure 3b. Careful observation of the peak locations at different temperatures revealed that there was a slight peak shift toward the higher diffraction angle (2θ) when the SPS temperature increased from 800 to 1200 °C. SPS processing at higher temperatures led to lattice contraction, which resulted in a peak shift in XRD toward a higher 2θ value. The highest peak shift, 0.36° toward the right, was observed for the Ti sample processed at 1200 °C SPS temperature. Further, an increase in SPS temperature to 1400 °C resulted in an XRD peak shift toward a lower 2θ value by 0.37°. Such a dramatic trend change in peak shift was associated with lattice expansion due to the formation of a small amount of nano TiC within the Ti matrix, which was non-detectable in XRD. Similar peak shifts towards the lower 2θ value were observed due to the formation of TiC resulting from the reinforcement of graphene platelets within the Ti matrix [31]. Due to extreme processing temperatures (1400 °C) during SPS, the dissolution of carbon atoms from the graphite foil, punch, and die resulted in the formation of the TiC reaction phase, which is further discussed in the following microstructure analysis section. All sintered samples exhibited a c/a ratio in the range of 1.58–1.59, which is in good agreement with the standard value of 1.588 (JCPDS Card No: 00-900-8517).

### 3.2. Microstructure and Densification Analysis

**Figure 4:**
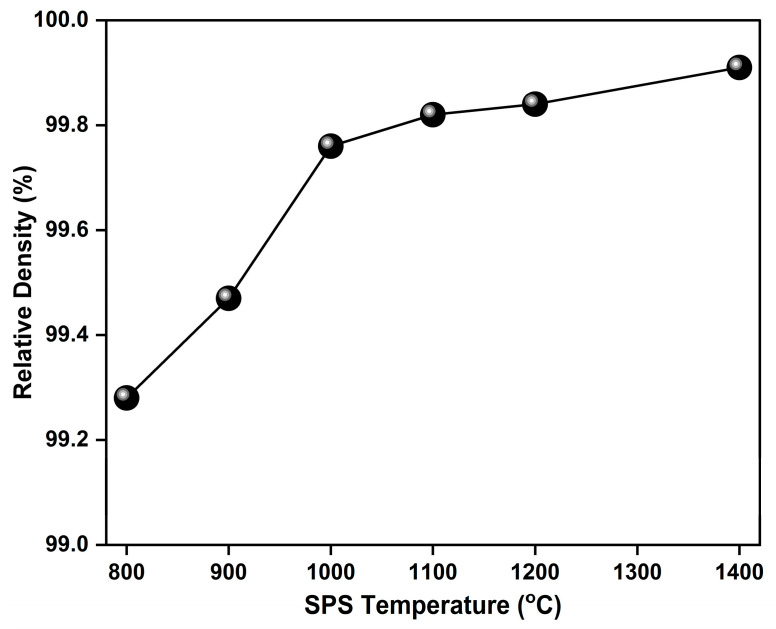
Variation in relative density with temperature.

**Figure 5:**
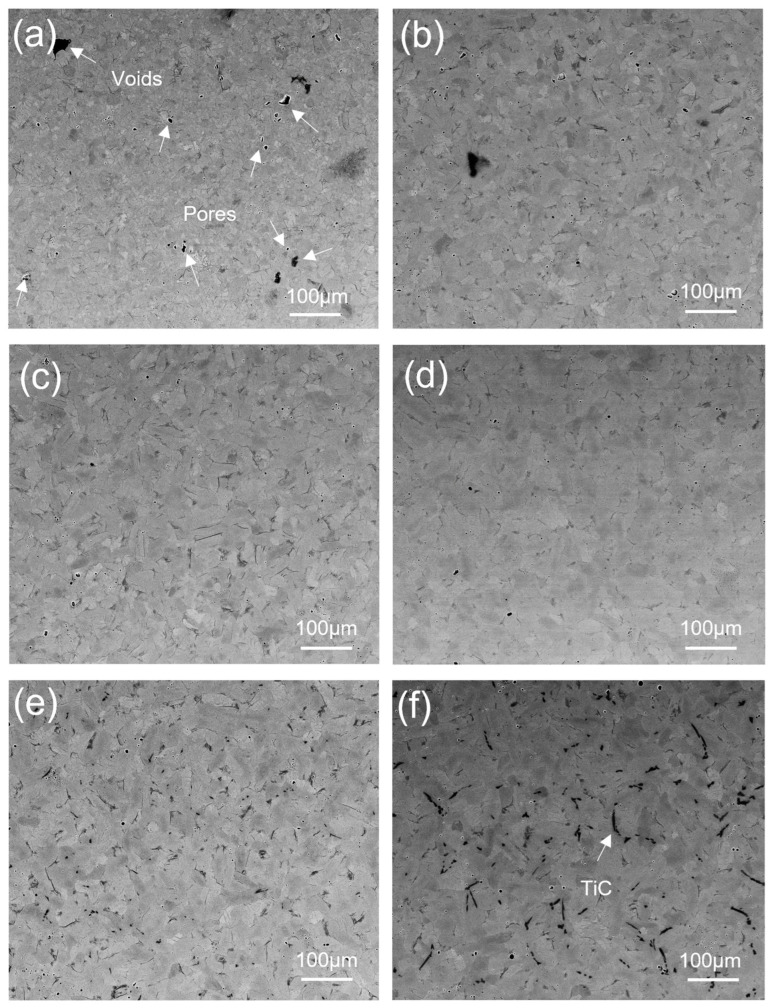
Backscatter SEM images of SPS-treated (unetched) pure Ti at 60 MPa and (**a**) 800 °C, (**b**) 900 °C, (**c**) 1000 °C, (**d**) 1100 °C, (**e**) 1200 °C, and (**f**) 1400 °C.

Figure 4 presents a plot depicting the relationship between relative density and sintering temperatures for all SPS sintered samples. The graph shows an upward trend in the relative density with an increase in sintering temperature. This clearly indicates that SPS temperature plays an important role in achieving full densification of samples. Backscattered SEM images of the SPS-processed Ti samples at different temperatures are shown in Figure 5. The substantial presence of fine porosity and voids in Figure 5a compared with Figure 5b–f reflects a decrease in porosity with an increase in temperature. The lowest relative density, at 800 °C, was 99.28%, whereas the highest density reached 99.91% at 1400 °C. The relative density of all SPS-processed Ti samples was higher than 99%, irrespective of SPS temperatures. The diffusion of different phases depends on temperature and time, and it can be effectively controlled in sintering. Pure titanium exhibits an α phase at room temperature. The phase transition of pure titanium into the β phase occurs at temperatures higher than 882 °C, known as β transus temperature. In pure titanium, the β-Ti phase has a higher diffusion coefficient than the α-Ti phase. So, a temperature above β transus increases diffusion, resulting in higher densification at higher temperatures [27]. The other contributing factors to higher densification are the fine particle size of the initial powder [32] and the densification mechanisms during sintering. There are four stages of sintering: activation and refining powder, neck formation, sintering neck growth, and plastic deformation and densification. The first two stages are due to the discharge of sparks through particles that break the oxide layer and form neck-on particles by overheating. In the third and fourth stages, current flows through the neck area and generates heat due to the joule effect that leads the thermal softening of powder to faster deformation and densification [33]. Applying a specific pressure and temperature in the third and fourth stages is crucial in controlling densification. For pure Ti, a temperature of 800 °C and pressure of 60 MPa are sufficient to achieve 99% relative density.

**Figure 6:**
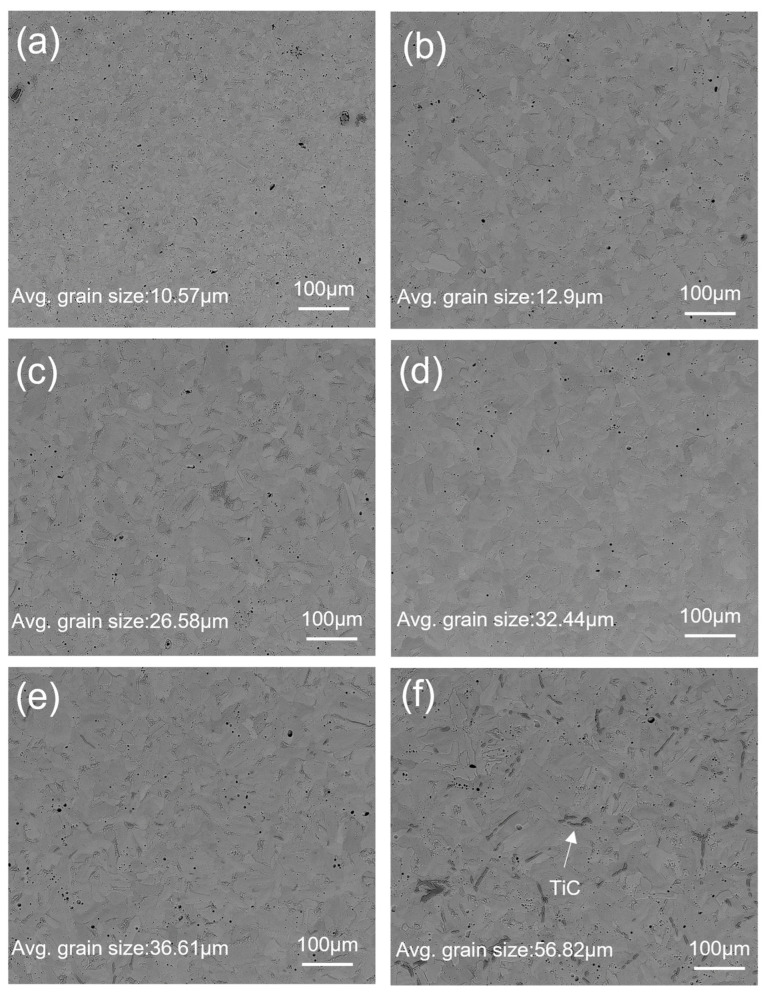
Backscatter SEM images of SPS-treated (etched) pure Ti at 60 MPa and (**a**) 800 °C, (**b**) 900 °C, (**c**) 1000 °C, (**d**) 1100 °C, (**e**) 1200 °C, and (**f**) 1400 °C.

Figure 5 and Figure 6 show the backscattered SEM images of sintered Ti samples at different temperatures before and after etching (with Kroll reagent for 10 s). The SPS-processed pure Ti samples exhibited polyhedral and equiaxed grain structures, and exhibited slight grain growth when the SPS temperature increased from 800 to 900 °C. A further increase in SPS temperature above 900 °C resulted in significant grain growth with a heterogeneous distribution of irregular columnar grain morphologies, which can be seen in Figure 5c–f and Figure 6c–f. SEM images of etched samples with similar magnifications were used to measure the grain size because the grains and grain boundaries were precisely visible in the etched samples compared with those without etching.

Grain analysis was performed using ImageJ software, with an average of 50 grains reported in Figure 6. The smallest grain size of 10.57 µm was measured for the sample sintered at 800 °C, whereas the largest grain size of 56.82 µm was measured for the sample sintered at 1400 °C. The trend of the grain growth was approximately flat from 800 °C to 900 °C and steeper from 900 °C to 1400 °C. Such transitions in microstructure and grain growth were associated with the recrystallization temperature (β-transus): the β-Ti phase was less dense than the α-Ti phase and promoted higher diffusion above the β transus temperature, resulting in higher grain growth above 900 °C [34].

**Figure 7:**
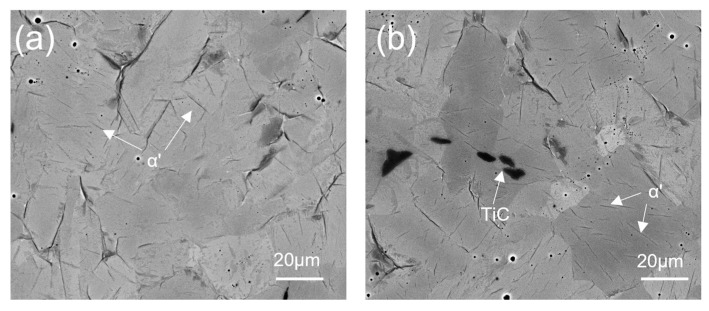
Higher-magnification backscatter SEM images of SPS-treated (unetched) pure Ti at temperatures of (**a**) 1200 °C and (**b**) 1400 °C.

Observing higher-magnification images of samples obtained under higher-temperature conditions revealed the needle-like substructure within each grain shown in Figure 7a,b. During rapid cooling after the sintering process, an incomplete transformation of the β-Ti phase to the α-Ti phase resulted in the formation of a fine needle-like morphology in the microstructure called α prime (α’). α’ is a similar martensitic phase typically obtained in steel and other non-ferrous alloys after rapid cooling [35,36]. α’ has the same crystal structure as α-Ti, and that is HCP. In general, such a transformation is observed in lean Ti alloys. Two types of α’ morphology were observed in past research: lath and plate [37]. Lath α’ is present in bundles of parallel fine needles. When the solute content increases, the lath α’ morphology is transferred into a single large-plate α’ morphology [38]. In Figure 7a,b, SEM images at higher magnifications show parallel bundles of fine α’ needles segregated in α-Ti grains, resulting in the formation of α + α’ microstructures, which may result in higher mechanical characteristics, as discussed in the next section.

**Figure 8:**
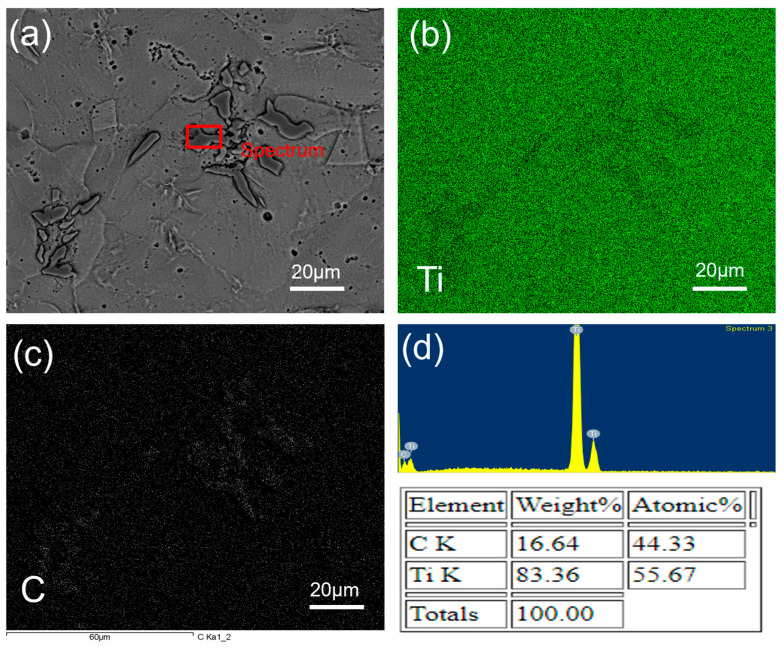
EDS Mapping of SPS-treated pure Ti at 60 MPa and 1400 °C; (**a**) SEM image, (**b**) Ti distribution, (**c**) carbon distribution, and (**d**) Ti and C peaks with each wt.%.

Figure 5e,f, Figure 6e,f, and Figure 7a,b show the formation of a new phase visible in dark black. In Figure 5e, Figure 6e, and Figure 7a, the intensity of the formation of the dark black phase is significantly lower than that observed in Figure 5f, Figure 6f, and Figure 7b. To confirm the presence of a dark phase, EDS mapping and point mapping were performed on pure Ti samples sintered at 1400 °C. Figure 8 shows the EDS mapping of the sample sintered at 1400 °C that clearly shows the presence of titanium (Figure 8b) and carbon (Figure 8c) in green and white area maps respectively. Point analysis of the dark black area on the same image for the same sample showed strong peaks of titanium and carbon, with approximately 45 at.% C and 55 at.% Ti (Figure 8d), suggesting the formation of the TiC phase at a sintering temperature of 1400 °C. The volume fraction of TiC phase in the sample sintered at 1200 °C was low and hence was not detected during EDS mapping.

### 3.3. Hardness and Tensile Property

**Figure 9:**
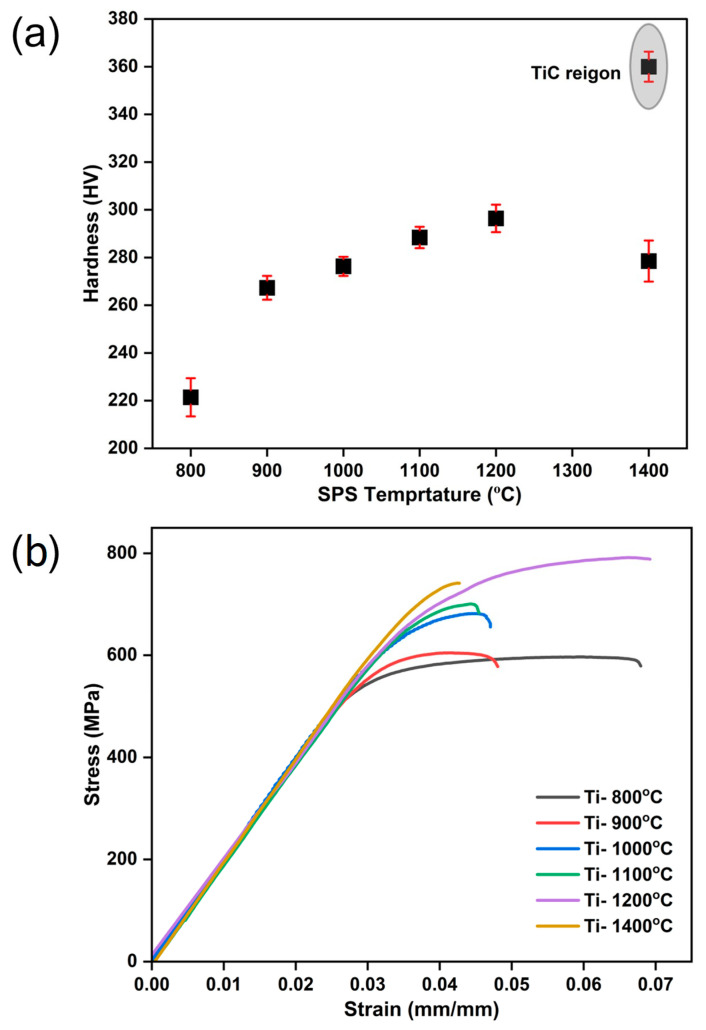
(**a**) Variation in hardness with temperature; (**b**) variation in tensile properties with temperature.

The variations in microhardness of the pure Ti samples with sintering temperature are shown in Figure 9a. The hardness of the sintered samples depended on the relative density, grain size, and formation of α’. The reported hardness values from the microhardness testing of samples sintered at 800, 900, 1000, 1100, and 1200 °C were 221.4, 267.3, 276.3, 288.4, and 296.4 HV respectively. An increase in sintering temperature from 800 to 1200 °C led to an increasing trend in hardness, primarily due to the higher relative density and formation of α’ phase. At 1400 °C, the sample showed hardness bifurcating in two regions: the first was the TiC-excluded region, and the second was the TiC-dominant region. The first region sample showed lower hardness, 278.5 HV, due to abnormal grain growth, resulting in lower restriction to dislocation movement during indentation loading. As TiC is a hard and brittle phase, in the second region, i.e., TiC-dominant region, indentation loading hinders the dislocation movement at the vicinity of the Ti–TiC interface [39], resulting in a significantly higher hardness of 360 HV than that of the first region and other temperature conditions. Finally, for the pure Ti sample, 1200 °C was the optimum sintering temperature for maximum uniform hardness to be achieved.

The tensile results of sintered pure Ti samples are plotted in Figure 9b. It shows the variation in the mechanical properties with sintering temperature. The yield strength (YS), ultimate tensile strength (UTS), and ductility data from the test are shown in Table 1. As the sintering temperature increased from 800 to 1200 °C, there were increases in YS and UTS, mainly due to the formation of the α’ phase and increasing relative density, which is in good agreement with the reported densification and microstructure details explained in the previous sections. For all samples, there was a slight variation in ductility between 4% and 7%. The highest ductility of 7% was observed at 1200 °C due to relatively larger grain size and higher densification. The degradation of mechanical properties, such as UTS and ductility, at the 1400 °C sintering temperature was mainly due to the formation of a large amount of TiC phase. The highest yield strength of 700 MPa was observed at 1400 °C, whereas the lowest yield strength of 488 MPa was observed at 800 °C. The highest ultimate tensile strength and ductility reported for sintered Ti samples at 1200 °C were 792 MPa and 7%, respectively. The statistical analysis results showed *p* < 0.05, confirming that there was an effect of sintering temperature on hardness and tensile strength.

### 3.4. Fractography Analysis

**Figure 10:**
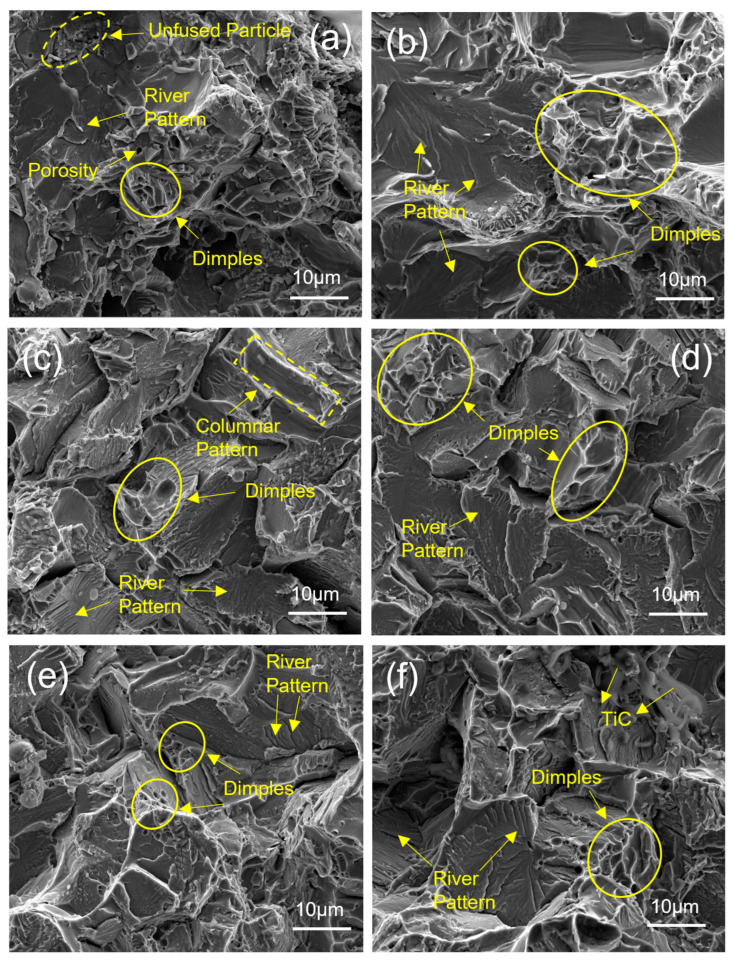
SEM images of fracture surfaces of SPS-treated pure Ti at 60 MPa and temperatures of (**a**) 800 °C, (**b**) 900 °C, (**c**) 1000 °C, (**d**) 1100 °C, (**e**) 1200 °C, & (**f**) 1400 °C after tensile test.

Figure 10 shows the SEM images of the fracture surface during tensile testing. Although high densification was achieved already at 800 °C, there were still fine pores in the sample, which can be seen in Figure 10a. This agrees with the previously mentioned densification in the relative density plot (Figure 4) and microstructure SEM image (Figure 5a). Such defects mainly occur due to incomplete particle fusion [40] at a sintering temperature of 800 °C. Partially fused particles are highlighted in Figure 10a. These often result in crack initiation and growth, causing lower mechanical strength in the sample during tensile testing (Figure 9b). Sintering temperatures above 800 °C resulted in no significant porosity in the fracture surface images in Figure 10b–f. This signifies the formation of complete bonding of the particles at higher temperatures. The presence of dimples and river-like patterns of radiating edges (tearing edges) are highlighted in Figure 10, reflecting the ductile and cleavage (brittle) fracture of the specimen during testing [41]. The fractured surface of the sintered sample changed from polyhedral and equiaxed to irregular columnar-like morphology when the SPS temperature was higher than 900 °C, which is in accordance with the observed trends in the microstructure explained in the previous section. In addition to this, Figure 10f shows traces of TiC. TiC is a hard and brittle intermetallic phase that behaves as a crack initiation site, resulting in the degradation of mechanical properties [31]. The formation of the TiC phase at 1400 °C was probably the only reason for lower ductility and UTS compared with sintering at 1200 °C.

## 4. Conclusions

Pure Ti samples were processed via Spark plasma sintering to investigate the effect of the sintering temperature range from 800 to 1400 °C at a constant pressure of 60 MPa and dwell time of 5 min in a controlled argon atmosphere. The density of the samples increased with increasing sintering temperatures. More than 99% densification was achieved with minor porosity for all temperatures ≥ 800 °C. Microstructure analysis depicted the formation of the fine α’ needles due to impurities in the as-received powder and rapid cooling during sintering, which increased with an increase in temperature. No significant difference in grain size was observed for the samples sintered at 800 °C and 900 °C, whereas higher grain growth was observed above 900 °C, mainly due to the higher diffusion coefficient of β-Ti above the recrystallization temperature. The formation of α’ contributed to achieving optimum hardness and tensile properties at 1200 °C. The formation of TiC at 1400 °C due to external impurities and higher grain growth during sintering was mainly responsible for the degradation of the mechanical properties.

## Figures and Tables

**Table 1:** Relative density, average grain size, hardness, YS, UTS, and elongation of pure Ti at different SPS temperatures.

SPS Temperature	Relative Density (%)	Avg. Grain Size (µm)	Hardness (HV)	YS (MPa)	UTS (MPa)	Elongation (%)
800 °C	99.28	10.57	221.4 ± 8.2	488 ± 10	597 ± 12	6.8 ± 0.2
900 °C	99.47	12.9	267.3 ± 5.4	518 ± 6	608 ± 10	4.8 ± 0.5
1000 °C	99.76	26.58	276.3 ± 4.3	592 ± 15	683 ± 8	4.7 ± 0.3
1100 °C	99.82	32.44	288.4 ± 4.5	610 ± 9	702 ± 13	4.5 ± 0.5
1200 °C	99.84	36.61	296.4 ± 5.7	642 ± 5	792 ± 11	7 ± 0.5
1400 °C	99.91	56.82	278.5 ± 8.6	700 ± 15	742 ± 7	4.2 ± 0.1

## Data Availability

The data presented in this study are available upon request from the authors.
